# Fixed-Point Fluid structure interaction analysis BASED ON geometrically exact approach

**DOI:** 10.1038/s41598-020-66854-5

**Published:** 2020-06-25

**Authors:** Mingliang Yu, Xueyuan Nie, Guowei Yang, Peinan Zhong

**Affiliations:** Key Laboratory for Mechanics in Fluid Solid Coupling Systems, Institute of Mechanics,Chinese Academy of Sciences, Beijing, 100190 China

**Keywords:** Mechanical engineering, Engineering

## Abstract

The fluid structure interaction analysis for structures exhibiting large deformations is carried out by using a strong coupling method, in which a fixed point method with Aitken’s dynamic relaxation is employed to accelerate convergence of the coupling iteration, and geometrically exact beam approach initiated by Simo is adopted to simulate the nonlinear flexible beam models. An improved implicit time integration algorithm is given to improve the computation accuracy of structural dynamics. To verify the validity of the fixed-point method in the compressible flows which is usually used in incompressible fluid, it is applied for flutter analysis of AGARD 445.6 wing in the transonic regime. The case of flow-induced vibration of a flexible beam demonstrates that the approach based on geometrically exact beam theory is suitable for the fluid structure interaction analysis and the fixed-point method with Aitken’s relaxation is of great efficiency and robustness in the FSI computation.

## Introduction

Fluid structure interaction (FSI) problems, which couple fluids to structures, are confronted in many fields such as civil engineering, especially in aeroelasticity. With an increased interest in the use of high-altitude-long-endurance (HALE) aircraft characterized by the long and slender wing structures, large displacements may occur at nominal operating conditions and as a result, new methods need to be developed to study the aeroelastic behaviors of such structures. The numerical simulations in the FSI computation of aeroelastic problems are mainly oriented towards two schemes: monolithic approach and the partition approach. The former solves the aerodynamic forces and structural motions simultaneously in the same manner with no need to distinguish between the fluid and structure part. It is possible to be implemented for simple structural problems with a few of degrees of freedom. The latter solves the aerodynamic forces and structural responses as separate modules in a different manner with fluid-structure interface to pass information between these domains. Because of using the existing methods for both parts independently, the partition approach is a favorite choice in current aeroelastic analysis.

The Dirichlet-Neumann partitioning^1^ using the existing solvers is one of partitioning techniques. In this approach, the nonlinear equations of fluid and structure domains are analysed by different solvers of subsystems. Partition approach usually adopts two different coupling strategies: loose coupling and strong coupling which are both commonly used in previous studies in computational aeroelasticity.

The loose coupling procedure is described as follows: (a) transfer the displacements on the interface boundary of the structure to the fluid system, (b) update the flow mesh accordingly and advance the fluid system to the next time step and compute the fluid traction forces, (c) convert the fluid interface forces into structural loads and advance the structure system to the next time step. Although this approach is easily realized and broadly used in the work^2–4^, it can obtain only first-order accuracy in time. To obtain a higher order accuracy in time, some other improved loose coupling algorithms which can get second-order accuracy in time are given, but the methods don’t enforce the synchronization of the two subsystems.

Besides the disadvantage of low order time accuracy, the loose coupling also shows instability and may introduce spurious solutions in simulation with large time step. Therefore, the strong coupling method is suggested. Yang *et al*.^5^ and Melville *et al*.^6^ described a strongly coupled algorithm in which aerodynamic loads were computed for each pseudo-time step and then structural response produced by those loads were computed, alternately. Though the fluid and structure systems can be perfectly synchronized at each time step with enough iterations, the computation is of high cost and the unsteady flow solver also needs to be modified.

Significant amount of research on aeroelasticity has focused on the structural motions governed by linear dynamic equations. But the analysis procedures based on small deformation assumption fail to analyze characteristics of the structures with large displacement. Block-Gauss-Seidel or fixed-point method^7–10^ is a strong coupling algorithm and commonly applied to FSI coupling between incompressible flow and structures with large deformation. It can be regarded as an extension of the loose coupling method. For each time step the computation of the two domains and the communication of interface motions and traction forces are repeated until interface displacements converge to the allowable tolerance to ensure the conservations of kinematics and dynamics. Some relaxation means can be employed because the convergence might be slow if the coupling of fluid and structure is strong^10^, such as flexible wings. Among those means, Aitken’s dynamic relaxation with robust stability and easy implementation is suggested in ref. ^1^.

The geometrically exact intrinsic beam model initiated by Hodges^11^ is widely adopted to compute the goemetric nonlinearity in aeroelasticity. Using this method, Hodges and Patil^12,13^ investigated the aeroelasticity of the wings of HALE aircraft. They pointed out that the wings of HALE aircraft would be highly deflected in flight and the effect of geometric nonlinearities on the flutter behavior was significant. It is frustrating for the method that the number of independent variables increases exponentially as the number of discrete nodes increases. Hence the geometrically exact beam model initiated by Simo^14^ is employed to study the nonlinear structural dynamics. In the numerical computation, the parameterization based on Euler angles is replaced by an implementation based on the use of quaternion parameters, which avoids singularity and minimizes storage requirements. In order to reduce the deviation when the integration time step is large, an improved Newmark algorithm based on implicit Simo-Newmark time stepping integration scheme is proposed.

The remainder of the paper is organized as follows: Firstly, the FSI coupling system, including the coupling conditions, coupling methods of time and space and the fixed-point method with Aitken’s dynamic relaxation, is presented. Secondly, the introduction of the geometrically exact beam theory follows, which describes the characteristics of nonlinear dynamics of slender structures. Finally, two numerical examples, which include the flutter analysis of AGARD 445.6 wing to verify the fixed-point method and the flow-induced vibration of a flexible beam to test the feasibility of geometrically exact beam method in FSI problems, are given and discussed in detail.

### Descriptions of FSI System

The interaction of the coupling system is essentially the communication of traction forces and motions on the interface of two subsystems^15^. Two partitions are processed by different programs with interaction effects treated as external vector inputs. To clarify the coupling of the problem, the Steklov-Poincare nonlinear equation^16^ is introduced to represent the coupling interaction on the interface.

Let ${\mathscr{F}}$ be the Dirichlet-to-Neumann (D-t-N) nonlinear fluid operator, also called Steklov-Poincare operator, mapping fluid displacements ***u***_*f*_ to interface traction forces ***λ***_*f*_ as follows,

1 $${{\boldsymbol{\lambda }}}_{f}={\mathscr{F}}({{\boldsymbol{u}}}_{f})$$

It represents the process of the deformation of the fluid domain and computation of flow field. Similarly, a Steklov-Poincare operator for the structural domain, relating structure displacements ***u***_*s*_ to interface traction forces ***λ***_*s*_ is express as

2 $${{\boldsymbol{\lambda }}}_{s}={\mathscr{S}}({{\boldsymbol{u}}}_{s})$$

Concerning the inverse of structure operator, we can define ${{\mathscr{S}}}^{-1}$ as a Neumann-to-Dirichlet (N-t-D) operator associating the interface traction forces and displacements of the structure

3 $${{\boldsymbol{u}}}_{s}={{\mathscr{S}}}^{-1}({{\boldsymbol{\lambda }}}_{s})$$

Generally the operators ${\mathscr{F}}$ and ${\mathscr{S}}$ do not have analytical expressions. The fluid domain is governed by compressible Euler/Navier-Stokes equations in aeroelasticity and the structural domain is solved by linear or nonlinear equations.

### Coupling conditions

The interface matching conditions in the following are based on two classical mechanics principles:

4 $${{\boldsymbol{u}}}_{f}={{\boldsymbol{u}}}_{s}={{\boldsymbol{u}}}_{\Gamma },{\rm{on}}\,\Gamma $$

5 $${{\boldsymbol{\lambda }}}_{f}+{{\boldsymbol{\lambda }}}_{s}=0,\,{\rm{on}}\,\Gamma $$

Eq.(4) represents the kinematic conservation which means the interface displacements of the two subsystems must be equivalent. Eq. (5) is the condition of dynamic conservation meaning that the resultant force on the interface must be zero. The fluid tractions are computed by

6 $${{\boldsymbol{\lambda }}}_{f}=-P\cdot {{\boldsymbol{n}}}_{f}+\tau \cdot {{\boldsymbol{n}}}_{f}$$

where *P*, $\tau$, ***n***_*f*_ represent the fluid pressure, viscid stress and normal vector on the fluid interface, respectively.

Applying Eq. (1) and Eq. (2) to dynamic coupling condition Eq. (5), we can get

7 $${\mathscr{F}}({{\boldsymbol{u}}}_{f})+{\mathscr{S}}({{\boldsymbol{u}}}_{s})=0$$

With kinematic coupling condition Eq. (4), Eq. (7) can also be stated based on interface displacements

8 $${\mathscr{F}}({{\boldsymbol{u}}}_{\Gamma })+{\mathscr{S}}({{\boldsymbol{u}}}_{\Gamma })=0$$

Using the definition of the inverse operator ${\mathscr{S}}$^−1^, we can express Eq. (8) as

9 $${{\mathscr{S}}}^{-1}({\mathscr{F}}({{\boldsymbol{u}}}_{\Gamma }))+{{\boldsymbol{u}}}_{\Gamma }=0$$

Let ${\mathscr{G}}$ be an operator defined by:

10 $${\mathscr{G}}={{\mathscr{S}}}^{-1}\circ (-{\mathscr{F}})$$

Eq. (9) can be reformulated using Eq. (10), resulting with the fixed-point formulation

11 $${\mathscr{G}}({{\boldsymbol{u}}}_{\Gamma })={{\boldsymbol{u}}}_{\Gamma }$$

So the standard algorithm to solve problem (11) is based on fixed-point iterations. The fixed-point method is to find interface displacement ***u***_Γ_ defined on Γ. One cycle of FSI iteration is denoted by the operator ${\mathscr{G}}$.

### Time coupling

In FSI problems, the loose coupled or Conventional Serial Staggered method (CSS)^17^ is broadly adopted, especially in computational aeroelasticity. At each time step, the D-t-N map ${\mathscr{F}}$ and N-t-D map ${{\mathscr{S}}}^{-1}$ are applyed only once. The procedure is as follows in Algorithm 1.

**Data:** start and end time(*t*_0_,*t*_max_), time step Δ*t* and initial interface displacement ${{\boldsymbol{u}}}_{\varGamma }^{0}$

**while** $$t < {t}_{\max }$$ do

Fluid solver: $${{\boldsymbol{\lambda }}}_{f}^{n+1}={\mathscr{F}}({{\boldsymbol{u}}}_{\Gamma }^{n})$$

Structure solver: $${{\boldsymbol{u}}}_{\varGamma }^{n+1}={{\mathscr{S}}}^{-1}(\,-\,{{\boldsymbol{\lambda }}}_{f}^{n+1})$$

$$t=t+\Delta t,n=n+1$$

end

**Algorithm 1:** CSS method

As mentioned in Section 1, this method is only first-order accuracy in time. Generalized Serial Staggered (GSS) method^18^ can improve the accuracy by prediction of the structure displacements at the beginning of the computation. The predictor is introduced

12 $${\mathscr{P}}({{\boldsymbol{u}}}_{\Gamma }^{n})={{\boldsymbol{u}}}_{\Gamma }^{n}+{\alpha }_{0}\Delta t{\dot{{\boldsymbol{u}}}}_{\Gamma }^{n}+{\alpha }_{1}\Delta t({\dot{{\boldsymbol{u}}}}_{\Gamma }^{n}-{\dot{{\boldsymbol{u}}}}_{\Gamma }^{n-1})$$

The prediction can get first-order time accuracy at ${\alpha }_{0}$ = 1 and ${\alpha }_{1}$ =0, and second-order time accuracy at ${\alpha }_{0}$ = 1 and ${\alpha }_{1}$ = 1/2. The GSS algorithm is described as follows in **Algorithm** 2,

**Data:** start and end time(*t*_0_, *t*_max_), time step Δ*t* and initial interface displacement ${{\boldsymbol{u}}}_{\Gamma }^{0}$

while $$t < {t}_{\max }$$ do

Predict displacements: $${{\boldsymbol{u}}}_{\Gamma ,p}={\mathscr{P}}({{\boldsymbol{u}}}_{\Gamma }^{n})$$

Fluid solver: $${{\boldsymbol{\lambda }}}_{f}^{n+1}={\mathscr{F}}({{\boldsymbol{u}}}_{\Gamma ,P})$$

Structure solver: $${{\boldsymbol{u}}}_{\Gamma }^{n+1}={{\mathscr{S}}}^{-1}(\,-\,{{\boldsymbol{\lambda }}}_{f}^{n+1})$$

$$t=t+\Delta t,n=n+1$$

end

**Algorithm 2:** Generalized Serial Staggered (GSS)

However, loose coupling strategy often leads to the so-called “added mass effect”^7^ causing computation instability, which highly depends on the mass ratio between fluid and solid with ${\rho }_{s}/{\rho }_{f}$ and the compressibility of the flow^9^. To avoid this drawback, the strong coupling algorithm called the block Gauss-Seidel (BGS) or fixed-point method is proposed. The standard fixed-point iteration with relaxation can be described as: for a given ***u***_*k*_, compute ${{\boldsymbol{u}}}_{k+1}={{\boldsymbol{u}}}_{k}+{\omega }_{k}({\bar{{\boldsymbol{u}}}}_{k}-{{\boldsymbol{u}}}_{k})$,where ${\bar{{\boldsymbol{u}}}}_{k}={\mathscr{G}}({{\boldsymbol{u}}}_{k})$ and ω_*k*_ is the relaxation coefficient. When ω_*k*_ = 1.0, there is no relaxation.

The fixed-point method can be expressed in terms of **Algorithm** 3.

**Data**: time step Δ*t*, and initial *t*_0_, final time t_,max_, initial interface displacement ${{\boldsymbol{u}}}_{\Gamma }^{0}$

**while** $$t < {t}_{\max }$$ do

Predict interface displacements: ${{\boldsymbol{u}}}_{\varGamma ,p}^{n+1}={\mathscr{P}}({{\boldsymbol{u}}}_{\varGamma }^{n})$

*k* = 0

**while** $$k < {k}_{\max }$$ do

FSI iteration: $${\bar{{\boldsymbol{u}}}}_{\Gamma ,k}^{n+1}={\mathscr{G}}({{\boldsymbol{u}}}_{\Gamma ,k}^{n+1})$$

Compute interface residual: ${r}_{k}^{n+1}={\bar{{\boldsymbol{u}}}}_{\Gamma ,k}^{n+1}-{{\boldsymbol{u}}}_{\Gamma ,k}^{n+1}$

Update the interface displacements ${{\boldsymbol{u}}}_{\Gamma ,k+1}^{n+1}={{\boldsymbol{u}}}_{\Gamma ,k}^{n+1}+{{\rm{\omega }}}_{k}{r}_{k}^{n+1}$

if $$\Vert {r}_{k}^{n+1}\Vert  < TOL$$ then

Break

else

*k* = *k* + 1

end

end$$t=t+\Delta t,n=n+1$$end

**Algorithm 3** Fixed-point method with relaxation

The key problem for this algorithm is how to determine the relaxation coefficient. One method called Aitken dynamic relaxation is suggested in Ref. ^1^. To illustrate this method, it is of great benefit to consider a scalar fixed-point equation

13 $$r(x)=f(x)-x=0,x\in R$$

This equation can be solved through the secant method. The iteration formula is: given ${x}_{k}$, ${x}_{k+1}$ is calculated by

14 $${x}_{k+1}={x}_{k}-{r}_{k}\frac{{x}_{k}-{x}_{k-1}}{{r}_{k}-{r}_{k-1}}$$

The Aitken relaxation coefficient is defined by

15 $${\omega }_{k}=\frac{{x}_{k}-{x}_{k-1}}{{r}_{k}-{r}_{k-1}}$$

Thus Eq. (14) becomes

16 $${x}_{k+1}={x}_{k}+{\omega }_{k}{r}_{k}$$

We compute the relaxation coefficient by

17 $${x}_{k+1}={x}_{k}+{r}_{k},{\omega }_{k+1}=-{\omega }_{k}\frac{{r}_{k}}{{r}_{k+1}-{r}_{k}}$$

Analogously, for the fixed-point Eq. (11), the interface residual is defined by

18 $${r}_{k}^{n+1}={\mathscr{G}}({{\boldsymbol{u}}}_{\Gamma ,k}^{n+1})-{{\boldsymbol{u}}}_{\Gamma ,k}^{n+1}$$

and the corresponding relaxation coefficient is calculated by

19 $${\omega }_{k+1}=-{\omega }_{k}\frac{{r}_{k}^{n+1}\cdot ({r}_{k+1}^{n+1}-{r}_{k}^{n+1})}{{\Vert {r}_{k+1}^{n+1}-{r}_{k}^{n+1}\Vert }^{2}}$$

For the first FSI cycle, it’s suggested to use the ${\omega }^{n}$ of the previous time step with a constrained parameter^1^

20 $${\omega }_{0}^{n+1}=\,\max ({\omega }^{n},{\omega }_{\max })$$

Therefore, the interface displacements of the next step are

21 $${{\boldsymbol{u}}}_{\Gamma ,k+1}^{n+1}={{\boldsymbol{u}}}_{\Gamma ,k}^{n+1}+{\omega }_{k}{{\boldsymbol{r}}}_{\Gamma ,k}^{n+1}$$

### Space coupling

Since different solvers are employed, the mesh on the interface of fluid and structural domains usually don’t match with each other. So an appropriate transfer scheme of two domains on the interface is important for the accuracy of the whole simulation. The transfer scheme must strictly maintain the conservation of momentum and energy as well as possess high efficiency and accuracy.

The equivalence of virtual work is considered to construct a conservative scheme^19^.

22 $$\delta W=\delta {{\boldsymbol{u}}}_{f}\cdot {\lambda }_{f}=\delta {{\boldsymbol{u}}}_{s}\cdot {\lambda }_{s}$$

Introduce the coupling matrix **H** mapping the structure displacements to aerodynamic ones,

23 $${{\boldsymbol{u}}}_{f}={\bf{H}}\cdot {{\boldsymbol{u}}}_{s}$$

And the relation of virtual displacements of fluid and structure mesh is achieved

24 $$\delta {{\boldsymbol{u}}}_{f}={\bf{H}}\cdot \delta {{\boldsymbol{u}}}_{s}$$

The structural traction ***λ***_*s*_ is calculated using Eq. (22) and Eq. (24).

25 $${\lambda }_{s}={{\bf{H}}}^{T}\cdot {\lambda }_{f}$$

Therefore, once the transfer matrix is determined, it can be used to calculate the aerodynamic nodes displacements and structural traction forces.

In the following, an interpolation scheme with Radial Basis Function (RBF) is presented. RBF is firstly proposed by Wendland and his coworkers^19,20^. The general form of interpolation based on RBFs is

26 $$s(x)=\mathop{\sum }\limits_{i=1}^{N}{a}_{i}\phi (\Vert x-{x}_{i}\Vert )+p(x)$$

where $\phi (\,\cdot \,)$ is a specific kind of RBF, *x*_*i*_ is coordinates of center points, *p*(*x*) is a polynomial.

Coefficients *a*_*i*_ and polynomials can be determined by the interpolation condition

27 $${d}_{a}=\mathop{\sum }\limits_{i=1}^{N}{a}_{i}\phi (\Vert {x}_{a}-{x}_{i}\Vert )+p({x}_{a})$$

and the additional condition

28 $$\mathop{\sum }\limits_{i=1}^{N}{a}_{i}q(\Vert {x}_{i}\Vert )=0$$

for all polynomials q with a degree deg(*q*) ≤ deg(*p*). The minimal degree of the polynomial depends on the choice of the basis function $\phi$. A unique interpolation that satisfies both Eqs. (27) and (28) is determined if the basis function $\phi$ is conditionally positive definite of order *m*. Only linear polynomials are used here

29 $$p(x)={\beta }_{0}+{\beta }_{1}x+{\beta }_{2}y+{\beta }_{3}z$$

In FSI, the number of fluid and structure nodes *N*_*f*_ and *Ns* on the interface are provided beforehand

30 $$\begin{array}{c}X=\{\begin{array}{cccc}{x}_{1}, & {x}_{2,} & \cdots  & {x}_{{N}_{s}}\end{array}\},\\ Y=\{\begin{array}{cccc}{y}_{1}, & {y}_{2,} & \cdots  & {y}_{{N}_{f}}\end{array}\}\end{array}$$

The points on the structure interface are taken as center points and their displacements are known as ***u***_*s*_. Using the interpolation condition Eq. (27) and additional condition Eq. (28), we calculate the coefficients by solving the equations

31 $$[\begin{array}{c}{{\boldsymbol{u}}}_{s}\\ 0\end{array}]=[\begin{array}{cc}{{\bf{C}}}_{ss} & {{\bf{P}}}_{s}\\ {{\bf{P}}}_{s}^{T} & 0\end{array}]\,[\begin{array}{c}\alpha \\ \beta \end{array}]$$

where $${{\bf{C}}}_{ss}=[\begin{array}{cccc}{\phi }_{{s}_{1}{s}_{1}} & {\phi }_{{s}_{1}{s}_{2}} & \cdots  & {\phi }_{{s}_{1}{s}_{{N}_{s}}}\\ {\phi }_{{s}_{2}{s}_{1}} & {\phi }_{{s}_{2}{s}_{2}} & \cdots  & {\phi }_{{s}_{2}{s}_{{N}_{s}}}\\ \vdots  & \vdots  & \ddots  & \vdots \\ {\phi }_{{s}_{{N}_{s}}{s}_{1}} & {\phi }_{{s}_{{N}_{s}}{s}_{2}} & \cdots  & {\phi }_{{s}_{{N}_{s}}{s}_{{N}_{s}}}\end{array}]$$, $${{\bf{P}}}_{s}=[\begin{array}{cccc}1 & {x}_{{s}_{1}} & {y}_{{s}_{1}} & {z}_{{s}_{1}}\\ 1 & {x}_{{s}_{2}} & {y}_{s2} & {z}_{{s}_{2}}\\ \vdots  & \vdots  & \vdots  & \vdots \\ {1}_{{s}_{{N}_{s}}{s}_{1}} & {x}_{{s}_{{N}_{s}}} & {y}_{{s}_{{N}_{s}}} & {z}_{{s}_{{N}_{s}}}\end{array}]$$, $${\phi }_{{s}_{i}{s}_{j}}=\phi (\Vert {x}_{{s}_{i}}-{x}_{{s}_{j}}\Vert )$$

The displacements of fluid nodes on thze interface are interpolated by

32 $${{\boldsymbol{u}}}_{f}=[\begin{array}{cc}{{\bf{M}}}_{fs} & {{\bf{P}}}_{f}\end{array}][\begin{array}{c}\alpha \\ \beta \end{array}]$$

where $${{\bf{M}}}_{fs}=[\begin{array}{cccc}{\phi }_{{f}_{1}{s}_{1}} & {\phi }_{{f}_{1}{s}_{2}} & \cdots  & {\phi }_{{f}_{1}{s}_{{N}_{s}}}\\ {\phi }_{{f}_{2}{s}_{1}} & {\phi }_{{f}_{2}{s}_{2}} & \cdots  & {\phi }_{{f}_{2}{s}_{{N}_{s}}}\\ \vdots  & \vdots  & \ddots  & \vdots \\ {\phi }_{{f}_{{N}_{s}}{s}_{1}} & {\phi }_{{f}_{{N}_{s}}{s}_{2}} & \cdots  & {\phi }_{{f}_{{N}_{s}}{s}_{{N}_{s}}}\end{array}]$$, $${{\bf{P}}}_{f}=[\begin{array}{cccc}1 & {x}_{{f}_{1}} & {y}_{{f}_{1}} & {z}_{{f}_{1}}\\ 1 & {x}_{{f}_{2}} & {y}_{{f}_{2}} & {z}_{{f}_{2}}\\ \vdots  & \vdots  & \vdots  & \vdots \\ 1 & {x}_{{f}_{{N}_{f}}} & {y}_{{f}_{{N}_{f}}} & {z}_{{f}_{{N}_{f}}}\end{array}]$$, $${\phi }_{{f}_{i}{s}_{j}}=\phi (\Vert {x}_{{f}_{i}}-{x}_{{s}_{j}}\Vert )$$

Combining Eqs. (31) and (32), ***u***_*f*_ is calculated as follows

33 $${{\boldsymbol{u}}}_{f}=[\begin{array}{cc}{{\bf{M}}}_{fs} & {{\bf{P}}}_{f}\end{array}]{[\begin{array}{cc}{{\bf{C}}}_{ss} & {{\bf{P}}}_{s}\\ {{{\bf{P}}}_{s}}^{T} & 0\end{array}]}^{-1}[\begin{array}{c}{{\boldsymbol{u}}}_{s}\\ 0\end{array}]=\hat{{\bf{H}}}[\begin{array}{c}{{\boldsymbol{u}}}_{s}\\ 0\end{array}]$$

Thus the matrix **H** is built by the first *N*_*f*_ rows and *Ns* columns of $\hat{{\bf{H}}}$

34 $${\bf{H}}=\hat{{\bf{H}}}[\begin{array}{cc}1:{N}_{f}, & 1:{N}_{s}\end{array}]$$

RBF interpolation can also be used to process mesh deformation. The procedure is similar to the transformation of interface displacements. Nodes on the moving boundaries are firstly selected as center points and the displacements of flow volume grid are interpolated. For large scale of data, a greedy algorithm is adopted to reduce the number of center points.

## Geometrically Exact Beam Theory

To investigate the geometric nonlinearity of slender flexible body, structure nonlinearities solver based on geometrically exact beam theory has been developed by Institute of Mechanics, Chinese Academy of Sciences for Structure dynamics computations. In this section, an introduction of geometrically exact beam theory is presented. For detailed descriptions, refer to the researches^21,22^. Throughout this section, *S* refers to the arc-length parameter of the rigid beam, and the prime (·)′ represents the derivative with respect to *S*. The dot $\mathop{(\,\cdot \,)}\limits^{\cdot }$ denotes the derivative with respect to time *t*. Let ${\{{e}_{i}\}}_{i=1}^{3}$ represent the standard fixed orthonormal basis in **R**^3^. The uppercase letter denotes material variable and the lowercase letter denotes spatial variable.

### Kinematics

**Figure 1:**
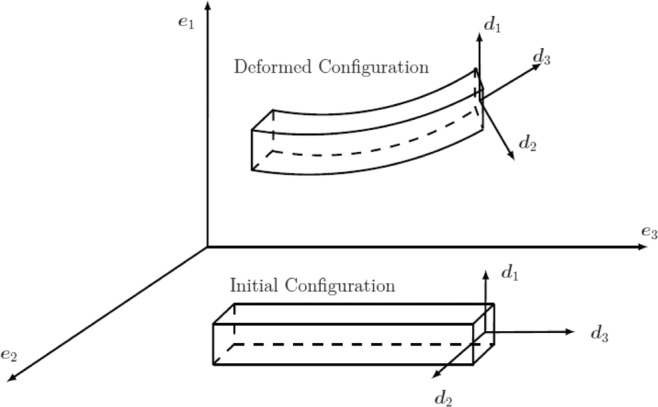
Basic kinematics of the beam.

Defining a family of cross-sections the centroids connected by the centerline of the beam, we can describe the configuration of a beam. The space curve ***φ***(*S*) is used to describe the centerline. The cross-sectional plane is determined by a triad of orthonormal basis ${\{{d}_{i}\}}_{i=1}^{3}$, which is attached to the cross-section and a moving frame. The normal vector ***d***_3_, as shown in Fig. 1 ^15^, is required to satisfy the condition ${d}_{3}(S)\cdot \varphi {\prime} (S) > 0$ which limits the amount of shearing and prevents beam configurations from degenerating.

For the sake of simplicity, the centerline of the undeformed beam is taken to be a straight line which coincides with the axis of ***e***_3_. Out of all possible configurations, the undeformed state is chosen to be the reference one. In this configuration, choose the triad ${\{{d}_{i}^{0}\}}_{i=1}^{3}$ with ${d}_{1}^{0}$ and ${d}_{2}^{0}$ directed along the principal axes of the cross-section and ${d}_{3}^{0}$ tangent to the curve ${\varphi }_{0}$. But it is not necessary that the vector field ***d***_3_ be tangent to the deformed centroid. Thus such model can describe the shear deformation.

The orthonormal frame ${\{{d}_{i}(S)\}}_{i=1}^{3}$ along the centerline relative to a fixed basis ${\{{e}_{i}\}}_{i=1}^{3}$ in **R**^3^ is uniquely specified by a map $S\mapsto {\boldsymbol{\Lambda }}(S)\in {\rm{SO}}(3)$, such that ${d}_{i}={\boldsymbol{\Lambda }}{e}_{i}$, where SO(3) represents the special orthogonal group or the rotation group. Compared with standard Cosserat beam theory, elements in SO(3) are regarded as the basic variables and make no further reference to directors field. Hence, a Cosserat beam configuration is completely determined by a pair of curves: $S\mapsto \varphi (S)\in {{\rm{R}}}^{3}$ and $S\mapsto \Lambda (S)\in {\rm{SO}}(3)$. The set ***Q*** of all possible configurations of the beam is described by

35 $${\bf{Q}}=\{{\boldsymbol{\Phi }}=(\varphi ,{\boldsymbol{\Lambda }})|\varphi :[0,L]\to {R}^{3},{\boldsymbol{\Lambda }}:[0,L]\to {\rm{SO}}(3)\}$$

### Governing equations

Let ***n***(*S*) and ***m***(*S*) denote the contact resultant force and moment respectively, acting on the cross-section at *S*^21^. On the Neumann boundary ${\partial }_{R}I$, **n** and **m** are specified with the values $\tilde{{\boldsymbol{n}}}$ and $\tilde{{\boldsymbol{m}}}$. On the Dirichlet boundary ${\partial }_{\varPhi }I$, $\varphi$ and ${\boldsymbol{\Lambda }}$ are specified with the values $\tilde{\varphi }$ and $\tilde{{\boldsymbol{\Lambda }}}$. $\bar{{\boldsymbol{n}}}(S)$, $\bar{{\boldsymbol{m}}}(S)$, ***p***(*S*) and ${\boldsymbol{\pi }}(S)$ represent the designated body force, moment per unit of the reference length, linear momentum and angular momentum, respectively. ***p***(*S*) and ***π***(*S*) are computed as follows

36 $$\begin{array}{c}{\boldsymbol{p}}={\rho }_{A}v\\ \pi ={i}_{\rho }\omega \end{array}$$

where ${\rho }_{A}$ is the mass per unit length of the beam and ***i***_ρ_ is the spatial time- dependent inertia tensor. The local forms of balance equations are defined as

37 $$\begin{array}{c}\dot{{\boldsymbol{p}}}={\boldsymbol{n}}{\boldsymbol{{\prime} }}+\bar{{\boldsymbol{n}}}\\ \dot{\pi }={\boldsymbol{m}}{\boldsymbol{{\prime} }}+{\boldsymbol{\varphi }}{\boldsymbol{{\prime} }}\times {\boldsymbol{n}}+\bar{{\boldsymbol{m}}}\end{array}$$

where ***n***′ and ***m***′ represent the derivative of n and m with respect to *S*, respectively, and the boundary conditions are

38 $$\begin{array}{c}(\varphi ,{\boldsymbol{\Lambda }})=(\tilde{\varphi },\tilde{{\boldsymbol{\Lambda }}})\,on\,{\partial }_{\varPhi }I\\ (n,m)=(\tilde{n},\tilde{m})\,on\,{\partial }_{R}I\end{array}$$

The strain measures are calculated by

39 $${\boldsymbol{\Gamma }}={{\boldsymbol{\Lambda }}}^{T}\varphi {\prime} -{{\boldsymbol{\Lambda }}}_{0}^{T}{{\varphi }_{0}}^{{\prime} },\,\hat{{\bf{K}}}={{\boldsymbol{\Lambda }}}^{T}{\boldsymbol{\Lambda }}{\boldsymbol{{\prime} }}-{{\boldsymbol{\Lambda }}}_{0}^{T}{{\boldsymbol{\Lambda }}{\boldsymbol{{\prime} }}}_{0}$$

Where $\hat{{\bf{K}}}$ denotes the skew matrix associating three dimensional vector **K** = (*K*_1_*;K*_2_*;K*_3_)

40 $$\hat{{\bf{K}}}=[\begin{array}{ccc}0 & {K}_{3} & -{K}_{2}\\ -{K}_{3} & 0 & {K}_{1}\\ {K}_{2} & -{K}_{1} & 0\end{array}]$$

${\boldsymbol{\Gamma }}$ is translational strain measure denoting the shear and extension strain, while **K** is rotational strain measure denoting the bending and torsional strain.

The constitutive law of the linear elastic material is expressed by the linear relations between the material contact force ${\bf{N}}={{\boldsymbol{\Lambda }}}^{T}n$ and material contact moment ${\bf{M}}={{\boldsymbol{\Lambda }}}^{T}m$, and the strain measures ${\boldsymbol{\Gamma }}$ and **K**,

41 $${\bf{N}}={{\bf{C}}}_{N}{\boldsymbol{\Gamma }},{\bf{M}}={{\bf{C}}}_{M}{\bf{K}}$$

where **C**_*N*_ and **C**_*M*_ are material elasticity tensors. When the beam cross-section is composed of a single isotropic material, **C**_*N*_ and **C**_*M*_ are

42 $$\begin{array}{c}{{\bf{C}}}_{N}=diag(G{A}_{1},G{A}_{2},EA),\\ {{\bf{C}}}_{M}=diag(E{I}_{1},E{I}_{2},GJ)\end{array}$$

*E* and *G* are the Young’s modulus and the shear modulus. *A* is the cross-section area of the beam, and *A*_1_, *A*_2_ are referred to the effective areas considering the cross-section distortion. *EA* represents the elastic axial stiffness of the cross-section, The terms *GA*_1_,*GA*_2_, *EI*_1_, *EI*_2_ and *GJ* are regarded as the shear stiffness, the elastic bending stiffness relative to principal axes ***d***_1_, ***d***_2_, and elastic torsional stiffness, respectively.

The weak form of the equilibrium equation is obtained by taking the dot product of Eq. (37) with an arbitrary admissible variation $(\eta ,\nu )$, which uses direct spatial integration by parts schemes over the domain [0, *L*]. As a result, it can be written as

43 $${\bf{G}}(\varphi ,{\boldsymbol{\Lambda }};\eta ,v)={{\bf{G}}}_{int}+{{\bf{G}}}_{dyn}-{{\bf{G}}}_{ext}=0$$

where **G**_*int*_ is the virtual work done by the stress caused by the elastic deformation of material, and given by

44 $${{\bf{G}}}_{int}={\int }_{0}^{L}[n\cdot (\eta {\prime} -v\times \varphi {\prime} )+m\cdot v{\prime} ]dS$$

***G***_dyn_ is the virtual work produced by the inertia of the beam, and computed by

45 $${G}_{dyn}={\int }_{0}^{L}(\dot{{\boldsymbol{p}}}\cdot {\boldsymbol{\eta }}{\boldsymbol{+}}\dot{{\boldsymbol{\pi }}}\cdot {\boldsymbol{v}})dS$$

and **G**_ext_ is the virtual works yielded by the externally applied loads and boundary stress resultants and obtained from the following expression:

46 $${G}_{ext}={\int }_{0}^{L}(\bar{{\boldsymbol{n}}}\cdot {\boldsymbol{\eta }}+\bar{{\boldsymbol{m}}}\cdot {\boldsymbol{v}})dS+[\tilde{{\boldsymbol{n}}}\cdot {\boldsymbol{\eta }}+\tilde{{\boldsymbol{m}}}\cdot {\boldsymbol{v}}]|{\partial }_{R}I$$

The procedure of the linearization of Eq. (43) can be found in^14^. And the interpolation of the configuration of beam is discussed in the paper of Zhong *et al*.^21^.

### Improved newmark integration algorithm

In the analysis of nonlinear structural dynamics, implicit schemes are usually taken in the time integration. In terms of computational efficiency, a large enough time step should be adopted. The Newmark integration algorithm is the most widely used method in the solving of structural dynamics. It is described as follows

47 $$\begin{array}{c}{\dot{{\boldsymbol{U}}}}_{n+1}={\dot{{\boldsymbol{U}}}}_{n}+[(1-\gamma ){\dot{{\boldsymbol{U}}}}_{n}+\gamma {\dot{{\boldsymbol{U}}}}_{n}+1]h\\ {{\boldsymbol{U}}}_{n+1}={{\boldsymbol{U}}}_{n}+{\dot{{\boldsymbol{U}}}}_{n}h+\left[\left(\frac{1}{2}-\beta \right){\dot{{\boldsymbol{U}}}}_{n}+\beta {\dot{{\boldsymbol{U}}}}_{n+1}\right]{h}^{2}\end{array}$$

where *h* represents the time step. When $\beta =\frac{1}{4}$ and $\gamma =\frac{1}{2}$, the Newmark method is second-order accurate in time and unconditionally stable. Note that the construction of the schemes are based on the linear dependence of ***U***_*n*+1_ on ***U***_*n*_, ${\dot{{\boldsymbol{U}}}}_{n}$, ${\ddot{{\boldsymbol{U}}}}_{n}$. For the centerline of the beam, there is nothing different with the classical scheme. However, the motion of the cross-section of the beam is described by a curve defined in the rotation group SO(3).

48 $$t\mapsto {\boldsymbol{\Lambda }}(S,t)\in {\rm{SO}}(3)$$

SO(3) is not linear space and the rotation matrix ${{\boldsymbol{\Lambda }}}_{n+1}$ cannot be estimated by a linear combination of ${{\boldsymbol{\Lambda }}}_{n}$, ${\dot{{\boldsymbol{\Lambda }}}}_{n}$, ${\ddot{{\boldsymbol{\Lambda }}}}_{n}$. Simo *et al*.^23^ proposed an implicit time stepping algorithm based on the discrete forms of the exponential map and parallel transport in the special orthogonal group SO(3). It performs well in the computation with a small time step but may result in considerable deviations from the exact solution with a large time step. Therefore, a modified Newmark algorithm is presented to improve the accuracy of the scheme given by Simo.

Firstly, Simo-Newmark algorithm is reviewed. For every rotation vector ***θ***, it can be mapped by the exponential map on the rotation matrix ${\boldsymbol{\Lambda }}\in {\rm{SO}}(3)$

49 $$\exp (\hat{{\boldsymbol{\theta }}})={1}_{3}+\frac{\sin \Vert {\boldsymbol{\theta }}\Vert }{\Vert {\boldsymbol{\theta }}\Vert }\hat{{\boldsymbol{\theta }}}+\frac{\sin (\Vert {\boldsymbol{\theta }}\Vert /2)}{2{(\Vert {\boldsymbol{\theta }}\Vert /2)}^{2}}{\hat{{\boldsymbol{\theta }}}}^{2}$$

where $\hat{{\boldsymbol{\theta }}}$ is the skew matrix associating the rotation vector ***θ***. This is known as Rodrigues’ formula.

Subscript *n* represents the temporal discrete approximate of time-varying variables at time *t*_*n*_, ***u***_*n*_ and ***θ***_*n*_ denote the incremental displacement and rotation vector from time *t*_*n*_ to time *t*_*n*+1_

50 $${{\boldsymbol{u}}}_{n}={{\boldsymbol{\varphi }}}_{n+1}-{{\boldsymbol{\varphi }}}_{n},{{\boldsymbol{\Lambda }}}_{n+1}=\exp (\hat{\theta }){{\boldsymbol{\Lambda }}}_{n}$$

The basic procedure of the discrete time stepping update is described by: given a configuration ${{\boldsymbol{\Phi }}}_{n}=({{\boldsymbol{\varphi }}}_{n},{{\boldsymbol{\Lambda }}}_{n})\in {\bf{Q}}$, linear and angular velocities $({{\bf{v}}}_{n},{{\boldsymbol{\omega }}}_{n})$, and linear and angular accelerations (***a***_*n*_, ***α***_*n*_), obtain the updated configuration ${{\boldsymbol{\Phi }}}_{n+1}=({\varphi }_{n+1},{{\boldsymbol{\Lambda }}}_{n+1})\in {\bf{Q}}$, corresponding accelerations (***a***_*n*+1_, ***α***_*n*+1_), in a manner that is consistent and stable with Eq. (43). The implicit time stepping method is represented in Table 1. For convenience, the relations of material and spatial variables are given in Table 2.

**Table 1:** Implicit time stepping algorithm.

Translation	Rotation
${{\boldsymbol{\varphi }}}_{n+1}={{\boldsymbol{\varphi }}}_{n}+{{\boldsymbol{u}}}_{n}$	${{\boldsymbol{\Lambda }}}_{n+1}={{\boldsymbol{\Lambda }}}_{n}\exp (\hat{{\boldsymbol{\Theta }}})=\exp (\hat{\theta }){{\boldsymbol{\Lambda }}}_{n}$
${{\boldsymbol{u}}}_{n}=h{{\boldsymbol{v}}}_{n}+{h}^{2}\left(\frac{1}{2}-\beta \right){{\boldsymbol{a}}}_{n}+{h}^{2}\beta {{\boldsymbol{a}}}_{n+1}$	${\hat{{\boldsymbol{\Theta }}}}_{n+1}=h{{\boldsymbol{\Omega }}}_{n}+{h}^{2}\left[\left(\frac{1}{2}-\beta \right){{\bf{A}}}_{n}\beta {{\bf{A}}}_{n+1}\right]$
${{\bf{v}}}_{n+1}={{\bf{v}}}_{n}+h[(1-\gamma ){{\bf{a}}}_{n}+\gamma {{\bf{a}}}_{n+1}]$	${{\boldsymbol{\Omega }}}_{n+1}={{\boldsymbol{\Omega }}}_{n}+h[(1-\gamma ){\bf{A}}+\gamma {{\bf{A}}}_{n+1}]$

**Table 2:** Relations between material and spatial representations of rotation.

Variable	Material	Spatial	Relation
Relative rotation vector	${{\boldsymbol{\Lambda }}}_{2}={{\boldsymbol{\Lambda }}}_{1}\exp ({\boldsymbol{\Theta }})$	${{\boldsymbol{\Lambda }}}_{2}=\exp ({\boldsymbol{\Theta }}){{\boldsymbol{\Lambda }}}_{1}$	$\theta ={\boldsymbol{\Lambda }}{\boldsymbol{\Theta }}$
Angular velocity	$\dot{{\boldsymbol{\Lambda }}}={\boldsymbol{\Lambda }}{\boldsymbol{\Omega }}$	$\dot{{\boldsymbol{\Lambda }}}=\omega {\boldsymbol{\Lambda }}$	$\omega ={\boldsymbol{\Lambda }}{\boldsymbol{\Omega }}$
Angular acceleration	$\dot{{\boldsymbol{\Omega }}}={\bf{A}}$	$\dot{\omega }=\alpha$	$\alpha ={\boldsymbol{\Lambda }}{\bf{A}}$

It is easy to get for the centerline

51 $${{\boldsymbol{v}}}_{n+1}=\frac{\gamma }{\beta h}{\boldsymbol{u}}+{{\boldsymbol{v}}}_{n+1}^{0},{{\boldsymbol{a}}}_{n+1}=\frac{1}{\beta {h}^{2}}{\boldsymbol{u}}+{{\boldsymbol{a}}}_{n+1}^{0}$$

where ${{\bf{v}}}_{n+1}^{0}$ and ${{\boldsymbol{a}}}_{n+1}^{0}$ depend on the configuration of the centerline at time *t*_*n*_

52 $$\begin{array}{c}{{\boldsymbol{a}}}_{n+1}^{0}=-\frac{1}{\beta h}{{\boldsymbol{v}}}_{n}-\left(\frac{1}{2\beta }-1\right){{\boldsymbol{a}}}_{n}\\ {{\boldsymbol{v}}}_{n+1}^{0}={{\boldsymbol{v}}}_{n}+h[(1-\gamma ){{\boldsymbol{a}}}_{n}+\gamma {{\boldsymbol{a}}}_{n+1}^{0}]\end{array}$$

And for the cross-sections, similar formulas are

53 $${{\boldsymbol{\Omega }}}_{n+1}=\frac{\gamma }{\beta h}{\boldsymbol{\Theta }}+{{\boldsymbol{\Omega }}}_{n+1}^{0},{{\bf{A}}}_{n+1}=\frac{1}{\beta {h}^{2}}{\boldsymbol{\Theta }}+{{\bf{A}}}_{n+1}^{0}$$

where ${{\boldsymbol{\Omega }}}_{n+1}^{0}$ and ${{\bf{A}}}_{n+1}^{0}$ are determined by

54 $$\begin{array}{c}{{\bf{A}}}_{n+1}^{0}=-\,\frac{1}{\beta h}{{\boldsymbol{\Omega }}}_{n}-\left(\frac{1}{2\beta }-1\right){{\bf{A}}}_{n}\\ {{\boldsymbol{\Omega }}}_{n+1}^{0}={{\boldsymbol{\Omega }}}_{n}+h[(1-\gamma ){{\bf{A}}}_{n}+\gamma {{\bf{A}}}_{n+1}^{0}]\end{array}$$

Given the incremental displacements of centerline $\Delta {{\boldsymbol{u}}}_{{\boldsymbol{n}}+1}^{{\boldsymbol{k}}}$ and material rotation vector of cross-section $\Delta {{\boldsymbol{\theta }}}_{n+1}^{k}$ from iteration *k* to *k* + 1 at time *t*_*n*+1_

55 $$\Delta {{\boldsymbol{u}}}_{n+1}^{k}={{\boldsymbol{u}}}_{n+1}^{k+1}-{{\boldsymbol{u}}}_{n}^{k},\Delta {{\boldsymbol{\theta }}}_{n+1}^{k}={{\boldsymbol{\theta }}}_{n+1}^{k+1}-{{\boldsymbol{\theta }}}_{n}^{k}$$

the update procedure of iterations is described in Table 3.

**Table 3:** Update procedure of the implicit time stepping algorithm.

Translation	Rotation
${{\boldsymbol{u}}}_{n+1}^{k+1}={{\boldsymbol{u}}}_{n+1}^{k}+\Delta {{\boldsymbol{u}}}_{n+1}^{k}$	${\hat{{\boldsymbol{\theta }}}}_{n+1}^{k+1}={\exp }^{-1}(\exp (\Delta {\hat{{\boldsymbol{\theta }}}}_{n+1}^{k})\exp ({\hat{{\boldsymbol{\theta }}}}_{n+1}^{k}))$
	$$\Delta {{\boldsymbol{\Theta }}}_{n+1}^{k}={{\boldsymbol{\Theta }}}_{n+1}^{k+1}-{{\boldsymbol{\Theta }}}_{n+1}^{k}={{\boldsymbol{\Lambda }}}_{n}^{T}\Delta {\theta }_{n+1}^{k}$$
${{\boldsymbol{\varphi }}}_{n+1}^{k+1}={{\boldsymbol{\varphi }}}_{n+1}^{k}+\Delta {{\boldsymbol{u}}}_{n+1}^{k}$	${{\boldsymbol{\Lambda }}}_{n+1}^{k+1}=\exp (\Delta {\hat{\theta }}_{n+1}^{k}){{\boldsymbol{\Lambda }}}_{n+1}^{k}={{\boldsymbol{\Lambda }}}_{n+1}^{k}\exp (\Delta {\hat{{\boldsymbol{\Theta }}}}_{n+1}^{k})$
${{\bf{v}}}_{n+1}^{k+1}={{\bf{v}}}_{n+1}^{k}+\frac{\gamma }{h\beta }\Delta {{\boldsymbol{u}}}_{n+1}^{k}$	${{\boldsymbol{\Omega }}}_{n+1}^{k+1}={{\boldsymbol{\Omega }}}_{n+1}^{k}+\frac{\gamma }{h\beta }\varDelta {{\boldsymbol{\Theta }}}_{n+1}^{k}$
${{\boldsymbol{a}}}_{n+1}^{k+1}={{\boldsymbol{a}}}_{n+1}^{k}+\frac{\gamma }{{h}^{2}\beta }\Delta {{\boldsymbol{u}}}_{n+1}^{k}$	${{\bf{A}}}_{n+1}^{k+1}={{\bf{A}}}_{n+1}^{k}+\frac{1}{{h}^{2}\beta }\varDelta {{\boldsymbol{\Theta }}}_{n+1}^{k}$

However, Makinen^24^ pointed out that there are some flaws in the Simo-Newmark scheme. It needs some modifications to improve the accuracy of time integration. Here we skip the derivation of the correction to the Simo-Newmark scheme and give the results directly. Let ${\boldsymbol{\Theta }}=\Vert {\boldsymbol{\Theta }}\Vert$ denote the Euclidean norm of the vector ${\boldsymbol{\Theta }}$ and an operator is defined by

56 $${\bf{T}}({\boldsymbol{\Theta }})={{\bf{1}}}_{3}+\frac{1-\,\cos \,{\boldsymbol{\Theta }}}{{{\boldsymbol{\Theta }}}^{2}}\hat{{\boldsymbol{\Theta }}}+\frac{{\boldsymbol{\Theta }}-\,\sin \,{\boldsymbol{\Theta }}}{{{\boldsymbol{\Theta }}}^{3}}{\hat{{\boldsymbol{\Theta }}}}^{2}$$

and the derivative of ${\bf{T}}({\boldsymbol{\Theta }})$ with respect to time *t* is

57 $$\dot{{\bf{T}}}({\boldsymbol{\Theta }},\dot{{\boldsymbol{\Theta }}})={j}_{1}\dot{\hat{{\boldsymbol{\Theta }}}}+{j}_{2}(\dot{\hat{{\boldsymbol{\Theta }}}}\hat{{\boldsymbol{\Theta }}}+\hat{{\boldsymbol{\Theta }}}\dot{\hat{{\boldsymbol{\Theta }}}})+({\boldsymbol{\Theta }}\cdot \dot{{\boldsymbol{\Theta }}})({j}_{3}\hat{{\boldsymbol{\Theta }}}+{j}_{4}{\hat{{\boldsymbol{\Theta }}}}^{2})$$

where *j*_1_, *j*_2_*,j*_3_, and *j*_4_ are functions of ${\boldsymbol{\Theta }}$ and are calculated by

58 $$\begin{array}{c}{j}_{1}=\frac{\cos \,{\boldsymbol{\Theta }}-1}{{{\boldsymbol{\Theta }}}^{2}},{j}_{2}=\frac{{\boldsymbol{\Theta }}-\,\sin \,{\boldsymbol{\Theta }}}{{{\boldsymbol{\Theta }}}^{3}},\\ {j}_{3}=\frac{2-2\,\cos \,{\boldsymbol{\Theta }}-{\boldsymbol{\Theta }}\,\sin \,{\boldsymbol{\Theta }}}{{{\boldsymbol{\Theta }}}^{4}},\\ {j}_{4}=\frac{-2{\boldsymbol{\Theta }}-{\boldsymbol{\Theta }}\,\cos \,{\boldsymbol{\Theta }}+3\,\sin \,{\boldsymbol{\Theta }}}{{{\boldsymbol{\Theta }}}^{5}}\end{array}$$

The modified implicit time stepping algorithm is described in Table 4, where ${\bf{T}}({{\boldsymbol{\Theta }}}_{n+1}^{k})$ and $\dot{{\bf{T}}}({{\boldsymbol{\Theta }}}_{n+1}^{k},{\dot{{\boldsymbol{\Theta }}}}_{n+1}^{k})$ are abbreviated as ${{\bf{T}}}_{n+1}^{k}$ and ${\dot{{\bf{T}}}}_{n+1}^{k}$, respectively. In the update of angular velocity, the function ${{\bf{T}}}_{n+1}^{k+1}$ is used to correct the incremental value $\Delta {{\boldsymbol{\Theta }}}_{n+1}^{k}$ compared with the Simo-Newmark algorithm. ${{\bf{T}}}_{n+1}^{k}$ and its derivative ${\dot{{\bf{T}}}}_{n+1}^{k}$ are used as an amendment to $\Delta {{\boldsymbol{\Theta }}}_{n+1}^{k}$ in the calculation of angular acceleration

**Table 4:** Improved Newmark algorithm.

Translation	Rotation
**Predictor phase**	
$$\begin{array}{c}{{\boldsymbol{u}}}_{n+1}^{0}=0\\ {a}_{n+1}^{0}=-\frac{1}{\beta h}{v}_{n}-\left(\frac{1}{2\beta }-1\right){a}_{n}\\ {v}_{n+1}^{0}={v}_{n}+h[(1-\gamma ){a}_{n}+\gamma {a}_{n+1}^{0}]\end{array}$$	$$\begin{array}{c}{{\boldsymbol{\theta }}}_{n+1}^{0}={{\boldsymbol{\Theta }}}_{n+1}^{0}=0\\ {\ddot{{\boldsymbol{\Theta }}}}_{n+1}^{0}=-\frac{1}{\beta h}{{\boldsymbol{\Omega }}}_{n}-\left(\frac{1}{2\beta }-1\right){{\bf{A}}}_{n}\\ {\dot{{\boldsymbol{\Theta }}}}_{n+1}^{0}={{\boldsymbol{\Omega }}}_{n}+h[(1-\gamma ){A}_{n}+\gamma {\ddot{{\boldsymbol{\Theta }}}}_{n+1}^{0}]\end{array}$$
Corrector phase for k = 0,1,…	
$$\begin{array}{cc}{{\boldsymbol{u}}}_{n+1}^{k+1}={{\boldsymbol{u}}}_{n+1}^{k}+\Delta {{\boldsymbol{u}}}_{n+1}^{k} & {\hat{{\boldsymbol{\theta }}}}_{n+1}^{k+1}=ex{p}^{-1}({\boldsymbol{e}}{\boldsymbol{x}}{\boldsymbol{p}}(\Delta {\hat{{\boldsymbol{\theta }}}}_{n+1}^{k}){\boldsymbol{e}}{\boldsymbol{x}}{\boldsymbol{p}}({\hat{{\boldsymbol{\theta }}}}_{n+1}^{k}))\\ & \Delta {{\boldsymbol{\Theta }}}_{n+1}^{k}={{\boldsymbol{\Theta }}}_{n+1}^{k+1}-{{\boldsymbol{\Theta }}}_{n+1}^{k}={{\boldsymbol{\Lambda }}}_{n}^{T}\Delta {{\boldsymbol{\theta }}}_{n+1}^{k}\end{array}$$	
$$\begin{array}{c}{{\boldsymbol{\varphi }}}_{n+1}^{k+1}={{\boldsymbol{\varphi }}}_{n+1}^{k}+\Delta {{\boldsymbol{u}}}_{n+1}^{k}{{\boldsymbol{\Lambda }}}_{n+1}^{k+1}=\exp (\Delta {\hat{{\boldsymbol{\theta }}}}_{n+1}^{k}){{\boldsymbol{\Lambda }}}_{n+1}^{k}\\ ={{\boldsymbol{\Lambda }}}_{n+1}^{k}\exp (\Delta {\hat{\varTheta }}_{n+1}^{k})\\ {v}_{n+1}^{k+1}={v}_{n+1}^{k}+\frac{\gamma }{\beta h}\Delta {{\boldsymbol{u}}}_{n+1}^{k}{{\boldsymbol{\Omega }}}_{n+1}^{k+1}={{\boldsymbol{\Omega }}}_{n+1}^{k}+\frac{\gamma }{h\beta }{{\bf{T}}}_{n+1}^{k+1}\Delta {{\boldsymbol{\Theta }}}_{n+1}^{k}\\ {a}_{n+1}^{k+1}={a}_{n+1}^{k}+\frac{1}{\beta {h}^{2}}\Delta {{\boldsymbol{u}}}_{n+1}^{k}{{\bf{A}}}_{n+1}^{k+1}={{\bf{A}}}_{n+1}^{k}+\left(\frac{1}{{h}^{2}\beta }{{\bf{T}}}_{n+1}^{k+1}+\frac{\gamma }{h\beta }{\dot{{\bf{T}}}}_{n+1}^{k+1}\right)\Delta {{\boldsymbol{\Theta }}}_{n+1}^{k}\end{array}$$	

## Numerical Examples

In the following FSI simulations, Stanford University Unstructured (SU2)^25,26^ designed for 2D and 3D CFD simulations, is a flow solver which solves the Reynolds-averaged Navier-Stokes(RANS) equations. An arbitrary Lagrangian-Eulerian (ALE) formulation of the NS equations can be written as follows:

59 $$\frac{\partial {\boldsymbol{u}}}{\partial t}+\nabla \cdot {{\bf{F}}}^{{\rm{c}}}({\boldsymbol{u}})-\nabla \cdot {{\bf{F}}}^{{\rm{v}}}({\boldsymbol{u}})=0$$

where **U** is a conservative variable defined as, ${\boldsymbol{u}}=({\rho }_{f},\rho {\bf{v}},\rho {\bf{E}})$

${{\bf{F}}}^{{\rm{c}}}({\boldsymbol{u}})\,{\rm{and}}\,{{\bf{F}}}^{{\rm{v}}}({\boldsymbol{u}})$ are convective fluxes and viscous fluxes, define by

60 $${{\bf{F}}}^{c}({\boldsymbol{u}})=[\begin{array}{c}{\rho }_{f}(v-{v}_{g})\\ {\rho }_{f}v\otimes (v-{v}_{g})+{\bf{I}}P\\ {\rho }_{f}E(v-{v}_{g})+{\rm{P}}v\end{array}]$$

61 $${{\bf{F}}}^{v}({\boldsymbol{u}})=[\begin{array}{c}0\\ \tau \\ \tau \cdot v+{\mu }_{{\rm{f}}}{{\rm{C}}}_{{\rm{p}}}\nabla T\end{array}]$$

where ${\rho }_{f}$, ***v*** = (v_1_, v_2_,v_3_), ***v***_g_, P, $\tau$ and ${\mu }_{f}$ represent the fluid density, the flow velocities in Cartesian coordinate system, the velocities of the nodes of the grid, the pressure, the viscous stress tensor and the fluid viscosity. *Cp* is the specific heat and **I** is a 3rd-order unit tensor. Specially, for inviscid flows the viscous fluxes disappear and the equations are reduced to Euler equations. Before solving of the equations, the new fluid grid have to be generated due to the moving boundaries. In SU2, the volumetric deformation is based on a classical spring method which may be computational costly and inefficient. So a fast multivariate interpolation method based on RBF with data reduction is applied to do the volumetric mesh deformation.

### AGARD 445.6 wing flutter analysis

**Figure 2:**
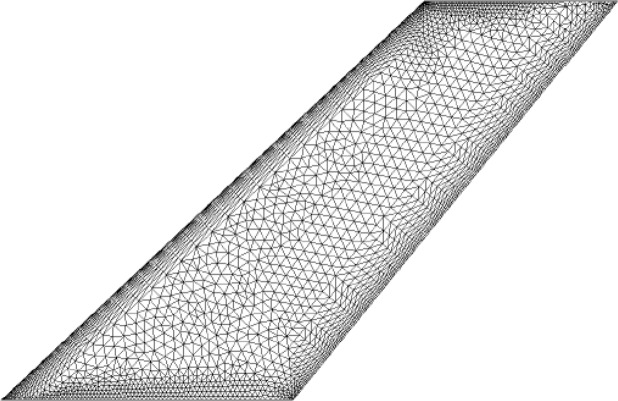
Surface mesh for the CFD computation.

To verify the validity of the fixed-point approach in the compressible fluid, which has been used in incompressible fluid, the AGARD 445.6 wing is selected in this section. It’s a standard test case with experimental data for transonic unsteady aerodynamic methods. The wing is a 45° swept-back wing with a NACA 65A004 airfoil section, an aspect ratio of 1.65, and a taper ratio of 0.66. The unstructured surface flow mesh is shown in Fig. 2., with 644,507 cells in the fluid domain and 4016 cells on the surface. The finite element model has been developed using 242 structural nodes for computation in the structural domain.

The unsteady compressible Euler equation with dual time stepping is used to solve unsteady flow. Roe-FDS scheme is selected for the evaluation of convective flux. Structural dynamics computations are performed by using the modal approach. The first four natural modes of AGARD 445.6 wing were given by Yates^27^. The modal dynamic equation are

62 $$\frac{d{q}_{i}}{d{t}^{2}}+{\omega }_{i}^{2}{q}_{i}={f}_{i},(i=1,\cdots ,4)$$

where ${q}_{i},{\omega }_{i}\,and\,{f}_{i}$ are the generalized displacement, natural frequency and generalized force of the *i*-th mode, respectively. These equations are solved by explicit Runge-Kutta method. The displacements of each structure node are calculated by

63 $${{\boldsymbol{u}}}_{s}(x,y,z,t)=\mathop{\sum }\limits_{i=1}^{4}{\Phi }_{i}(x,y,z){q}_{i}(t)$$

where ${\Phi }_{i}$ is the mode shape.

Introducing nondimensional mass ratio $\mu$, wing flutter boundary can be expressed by the reduced velocity ${V}^{\ast }$ as

64 $${V}^{\ast }=\frac{{{\boldsymbol{u}}}_{\infty }}{b{\omega }_{\alpha }\sqrt{\mu }}$$

where ${{\boldsymbol{u}}}_{\infty }$, b and ${\omega }_{\alpha }$ are the free stream velocity, the half-chord length of the root and the first torsion modal frequency, respectively.

**Figure 3:**
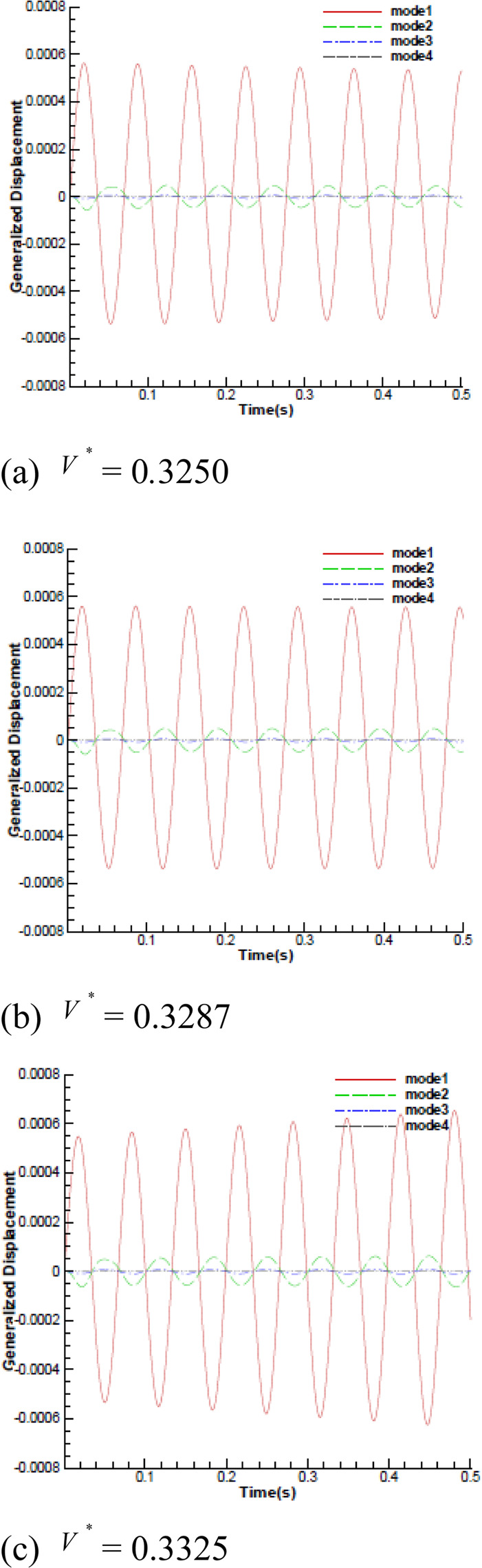
Time histories of generalized displacements at Ma = 0.96 with different reduced velocity.

**Figure 4:**
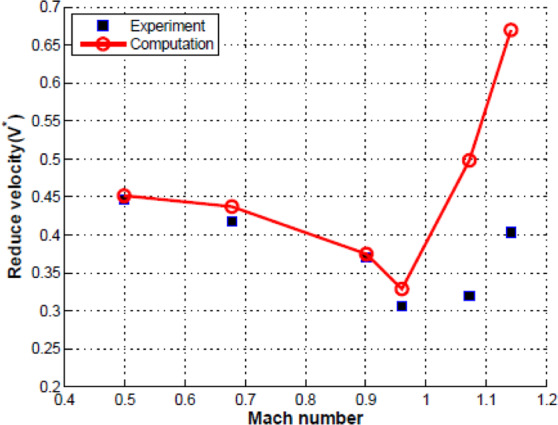
Flutter boundary for AGARD 445.6 wing.

The FSI was performed through fixed-point iteration with Aitken’s relaxation with the time step size of $5\times {10}^{-4}$ s. Initial analysis provided the steady-state flow field with the rigid wing in it. The time histories of the generalized displacements in all four modes that developed in response to aerodynamic forces are shown in Fig. 3 with different reduced velocity V^*^ at *Ma* = 0.96 and the angle of attack of 0 degree. With the different ${{\boldsymbol{u}}}_{\infty }$, several free stream static pressure and static temperature would be changed at a given Mach number. It can be seen that the all modal displacement amplitudes are in a decreasing trend when ${V}^{\ast }$ is smaller than the flutter boundary. With the value of ${V}^{\ast }$ close to the critical value, the aeroelastic equilibrium response is achieved. For ${V}^{\ast }$ bigger than the flutter boundary, the all modal displacement amplitudes show an increasing trend to the diverging response. At *Ma* = 0.96, the predicted flutter boundary value is 0.3287, which is close to the experimental result 0.3059 provided by ref. ^27^. The flutter speed for Mach 0.499 to 1.072 for the current method and the experimental results are shown in Fig. 4. On the whole, the numerical results at subsonic and transonic regime agree well with the experimental data. However, the flutter boundary values are higher in the computational solutions than the experimental ones in the supersonic regime. This difference between the predicted and measure results has also been noted in the previous studies, which is commonly believed that there exist measurement errors in the experiment of supersonic regime.

### Flow- induced vibration of a flexible beam

**Figure 5:**
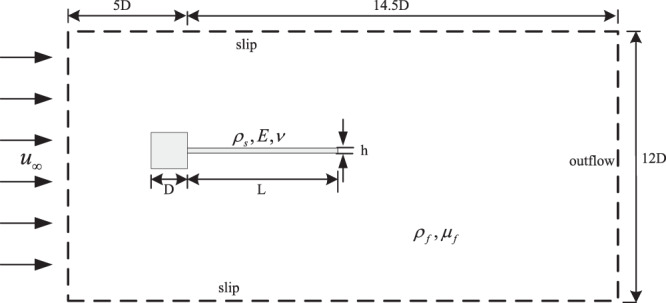
Flexible beam fixed to a square bluff body.

To demonstrate the ability of geometrically exact beam to deal with flexible structure in the FSI application, we consider a slender elastic beam attached to a square rigid body immersed in the flow. The geometry and the boundary conditions are shown in Fig. 5, where *D* = 1 m is the characteristic size of the square body, *L* = 4 m and *h* = 0.06 m are the length and thickness of the beam, respectively. The boundary conditions are set as: no-slip along the square body and the beam, slip walls on the upper and lower boundaries, inlet on the upwind side and outlet on the downwind side.

The fluid parameters are ${\rho }_{f}=1.18\times {10}^{-3}\,kg/{m}^{3}$ for the density and ${\mu }_{f}=1.82\times {10}^{-4}\,kg/m\cdot s$ for the viscosity. The inflow velocity is chosen as ${{\boldsymbol{u}}}_{\infty }=51.3\,m/s$. The Reynolds number is obtained as ${R}_{e}={\rho }_{f}D{{\boldsymbol{u}}}_{\infty }/{\mu }_{f}=332$. The material parameters of the solid are as, respectively, ${\rho }_{s}=0.1\,kg/{m}^{3}$ for structure density, $E=2.5\times {10}^{6}\,Pa$ for Young modulus and $\nu =0.35$ for Poisson ratio coefficient. The first natural frequency of the problem *f*_1_ = 3.03 Hz.

The fluid domain was solved through unsteady compressible Navier-Stokes equations with dual time method. For the time integration of the structural computation, the improved Newmark method described in Section 2 was adopted and SU2 was also used as the fluid solver.

**Figure 6:**
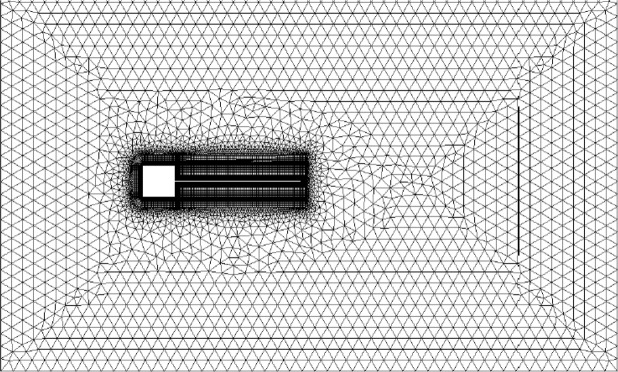
Fluid mesh for a flexible beam fixed to a square body.

This application originally carried out in^28^ has been used as a benchmark for FSI problem. A hybrid mesh was generated for CFD computation as shown in Fig. 6. To capture the vortex shedding accurately, refined quadrilateral elements were generated in the boundary layers and triangular elements are generated for the rest. The number of total points was 18984 and the number of total cells was 23872. The flexible beam was modeled by geometrically exact beam and discretized into 41 nodes and 40 finite element cells.

**Figure 7:**
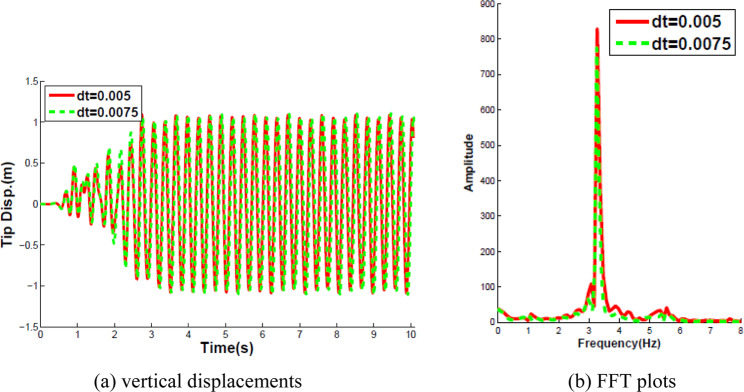
Curves of the vertical displacements of the oscillating tip of the beam in time and frequency.

The FSI problem was solved with dynamic Aitken’s relaxation besides using the second-order predictor to estimate the interface displacements. The Aitken relaxation technique was used with an initial value of 0.5, which rapidly increased to the range of values from 0.8 to 1.0. The convergence tolerance of the interface displacement residual, which is depicted as *TOL* in Algorithm 3, is set to ${10}^{-7}$. The comparisons of the displacement at the free end of the beam and corresponding Fourier analysis at the time steps Δ*t*_1_ = 0.005 s and Δ*t*_2_ = 0.0075 s are plotted in Fig. 7.

The following observations were made from the Fig. 7. The deviation of the responses obtained for Δ*t*_1_ and Δ*t*_2_ is very small. At the beginning of the simulation, the flow field was in a symmetric state and the cantilever was stationary. Then the flow exhibited an unsteady behavior with vortices shedding from the corner of the square body to induce oscillations of the flexible beam. After 3 seconds, the whole system reached to an almost harmonic response dominated by the vibration frequency of 3.2 Hz, close to the first eigenfrequency of the structure and the amplitude of the response is about 1.05. The amplitude and the dominant frequency of the response obtained both agree well with the previous results from the literature^26,28,29^.

**Figure 8:**
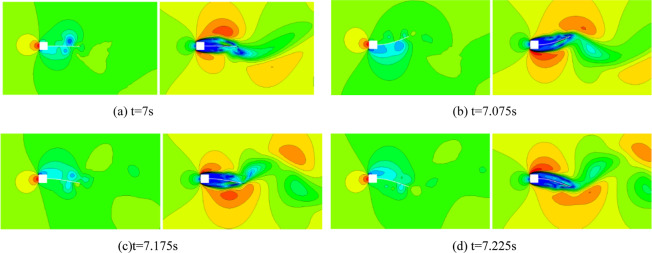
Pressure and velocity distributions of flow induced vibration of the flexible beam at Δ*t*_1_ = 0.005 s.

The flow pressure and velocity distributions at several instants during stable oscillations are displayed in Fig. 8. The deformed shapes of the flexible beam given by Fig. 8 reveal the vibration dominated by the first mode.

**Figure 9:**
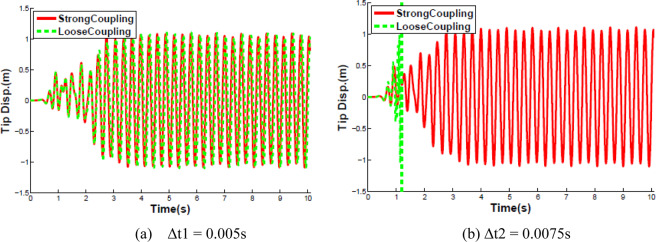
Verticals displacement at the tip of the beam with different coupling schemes.

The diagrams in Fig. 9 display the evolution of the tip displacement of the beam in time considering different coupling methods. Figure 9(a) shows that the responses obtained by loose coupling and by strong coupling deviate only by a small amount at the time step Δ*t*_1_ = 0.005 s. Contrary to Δ*t*_1_ = 0.005 s, the simulation from the loose coupling diverges due to added mass effect after hundreds of time steps though the convergence of the tip displacement obtained by the strong coupling is observed be good at Δ*t*_2_ = 0.0075 s. The results confirm the fact that the loose coupling method is more sensitive to added mass effect and diverges more easily with the increase of the time step size. Thus the use of the strong coupling method is justified and efficient to solve the coupled problem with the larger time step size.

**Figure 10:**
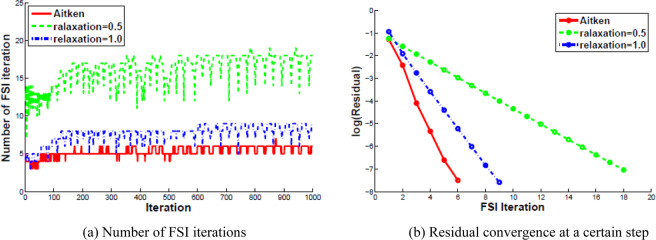
Comparison of Convergence rate with different relaxation methods.

The final study is considered with respect to different relaxation methods. The number of each FSI iterations is shown in Fig. 10(a). It can be seen that no more than 8 cycles in FSI iterations are required to reach the tolerance *TOL* = ${10}^{-7}$ with Aitken’s dynamic relaxation, nevertheless, methods with a fixed relaxation parameter $\omega =0.5$ and no relaxation $\omega =1.0$ lead to more iterations, especially a noticeable increase in fixed relaxation. Figure 10(b) presents the residual convergence for different relaxation methods. With constant $\omega$, a constant decrease of the residual with a low order is observed. With Aitken’s relaxation, the convergence exhibits a less smooth trend, with a faster decrease of the residual. Hence, Aitken’s dynamic relaxation is of great efficiency to speed up the FSI computation.

## Conclusions

In the work, we consider the fluid-structure interaction as the model problem, and discuss its solution procedure from the point view of finding the solution of the interface Steklov-Poincare equation. The computation is performed by the fixed-point method with Aitken’s dynamic relaxation. The open source CFD code SU2 is extended with the fixed-point method and integrated with two different structure solvers: the linear modal solver and the nonlinear dynamics solver based on geometrically exact beam theory of Simo. Numerical examples are provided to give evidence of the method effectiveness and efficiency. The case of AGARD 445.6 wing is computed by modal approach and proves the applicability of the fixed-point method in the analysis of transonic flow. The problem of flow-induced vibration of a flexible beam is handled by the fixed-point with Aitken’s relaxation in the strong coupling scheme. The strategy exhibits very good convergence properties in FSI iterations, as well as a sufficiently robust performance, especially in a relatively large time step size.
